# Electrical stimulation for chronic non-specific low back pain in a working-age population: a 12-week double blinded randomized controlled trial

**DOI:** 10.1186/1471-2474-14-117

**Published:** 2013-03-28

**Authors:** Matthew S Thiese, Matthew Hughes, Jeremy Biggs

**Affiliations:** 1 Rocky Mountain Center for Occupational & Environment Health, Department of Family and Preventive Medicine, University of Utah, 391 Chipeta Way, Suite C, Salt Lake City, UT, 84108, USA

**Keywords:** Chronic low back pain, Transcutaneous electrical nerve stimulation, Double-blind randomized controlled trial, H-wave, TENS, Usual care

## Abstract

**Background:**

Non-invasive electrotherapy is commonly used for treatment of chronic low back pain. Evidence for efficacy of most electrotherapy modalities is weak or lacking. This study aims to execute a high-quality, double-blinded randomized controlled clinical trial comparing 1) H-Wave® Device stimulation plus usual care with 2) transcutaneous electrical nerve stimulation (TENS) plus usual care, and 3) Sham electrotherapy plus usual care to determine comparative efficacy for treatment of chronic non-specific low back pain patients.

**Methods/Design:**

Patients- Chronic non-specific low back pain patients between ages of 18–65 years, with pain of at least 3 months duration and minimal current 5/10 VAS pain. Patients will have no significant signs or symptoms of lumbosacral nerve impingement, malignancy, spinal stenosis, or mood disorders.

Study design- Double blind RCT with 3 arms and 38 subjects per arm. Randomization by permuted blocks of random length, stratified by Workers Compensation claim (yes vs. no), and use of opioids. The null hypothesis of this study is that there are no statistically significant differences in functional improvement between treatment types during and at the end of a 12-week week treatment period.

Data collection- Subjective data will be collected using Filemaker Pro™ database management collection tools. Objective data will be obtained through functional assessments. Data will be collected at enrollment and at 1, 4, 8, and 12 weeks for each participant by a blinded assessor.

Interventions- H-Wave® device stimulation (Intervention A) plus usual care, transcutaneous electrical nerve stimulation (TENS) (Intervention B) plus usual care, and sham electrotherapy plus usual care (control). Each treatment arm will have identical numbers of visits (4) and researcher contact time (approximately 15 hours).

Outcomes- Primary outcome measure: Oswestry Disability Index. Secondary measures include: Rowland Morris Instrument, VAS pain score, functional evaluation including strength when pushing and pulling, pain free range of motion in flexion and extension. Outcome measures assessed at baseline, 1, 4, 8, and 12 weeks. Treatment failure will be defined if patient terminates assigned treatment arm for non-efficacy or undergoes invasive procedure or other excluded cointerventions. Data will be analyzed using intention-to-treat analysis and adjusted for covariates related to LBP (e.g. age) as needed.

**Discussion:**

Study strengths include complex randomization, treatment group allocation concealment, double blinding, controlling for co-interventions, rigorous inclusion criteria, assessment of compliance, plans for limiting dropout, identical assessment methods and timing for each treatment arm, and planned intention-to-treat analyses.

## Background

Low Back Pain (LBP) is one of the most common reasons for medical visits to physician offices and emergency departments, with an estimated 61 Million visits in 2007, up from an estimated 15 million visits in 1990 [1-3]. Chronic low back is associated with significant comorbidity and direct health care costs due to increased health care utilization [4]. The economic burden of low back pain is estimated between $20 and $50 billion annual cost to the U.S. economy [5]. Additionally, low back injuries result in an estimated 149 million lost workdays per year and an additional $28 billion in productivity losses [6]. Despite the relative pervasiveness of low back pain, and the enormous financial burden on the health care system, chronic low back pain remains a difficult condition to treat [7,8]. Identification of efficacious noninvasive, nonpharmacologic therapies could yield meaningful gains and result in substantial population improvement in morbidity and costs associated with LBP. A well-designed randomized trial is needed to determine comparative treatment efficacy of electrical stimulation treatments that are relatively inexpensive, noninvasive and nonpharmacologic.

Myriad treatments have been described for chronic LBP, yet the body of quality randomized trials demonstrating efficacy for many commonly used treatments is limited [9-11]. A recent evidence based review demonstrated that only 47 of 148 treatment interventions for Chronic LBP have quality randomized trials as the basis of recommendation for the intervention for low back pain [8]. Thus, the majority of commonly used interventions have little or no evidence of efficacy.

Specific to electrotherapy modalities, multiple devices producing varying electronic waveform types, wave frequency, and wave amplitude are used for analgesic relief of chronic pain conditions, including nonspecific low back pain. An evidence based review of electrotherapy literature identified a modest number of moderate and low quality trials that demonstrated evidence of transcutaneous electrical nerve stimulation (TENS) efficacy for select patients with chronic low back pain, however there is still no consensus regarding efficacy of TENS for treating chronic LBP [8,12-20]. Additionally, the reviews found insufficient quality evidence for recommending for or against the use of high-voltage galvanic therapy, interferential therapy, iontophoresis, percutaneous electrical nerve stimulation (PENS), microcurrent electrical stimulation, and sympathetic electrotherapy for treating chronic low back pain. Similarly, although there is strong anecdotal support, there are no reported quality randomized controlled trials demonstrating efficacy of the H-Wave® Device for symptomatic relief of chronic low back pain. Regardless of the evidence of efficacy of TENS in treating chronic LBP, it is a common modality for treating LBP due to high demand for noninvasive, nonpharmacologic interventions. It is highly prescribed due to low cost and low occurrence of side effects [21]. Identification of electrotherapy treatments with quality evidence of efficacy, low cost, and low adverse effect profile would be expected to result in improved patient function, reduced pain, reduced morbidity, improved productivity, and overall lower health care costs. In this study, we evaluate anecdotal properties of the H-Wave ® device, which is reported to be highly efficacious for many patients, and compares both economically and in safety with achieved TENS treatment protocols.

The H-Wave® Device (Electronic Waveform Lab, Inc) stimulation is an electrotherapy modality. The primary difference between other electrotherapies and H-Wave® is the waveform, with larger pulse durations and uniquely low frequencies. H-Wave® utilizes a bipolar exponentially decaying pulse with frequencies between 1-70Hz and a pulse duration of 5 milliseconds along with some other proprietary factors. The H-Wave® device has received FDA clearance for a number of indications, including home-based treatment of chronic pain, post-surgical pain, circulation, range of motion, and muscle spasms. The mechanism(s) of action for the H-Wave® Device are unique and include nitric-oxide dependent enhanced microcirculation and angiogenesis, stimulation and contraction of muscle fibers within the subdermal lymphatic system, and deactivation of the sodium pump within the nerve fiber [22,23]. While the exact mechanism of LBP treatment is unknown there are multiple mechanisms, including the one that is referenced above that may demonstrate efficacy for treating LBP. Other mechanisms include stimulation of nocireceptors and other nerve types to mask pain and allow patients to improve function.

The intent of this trial is to execute a high-quality^a^ double-blinded randomized controlled clinical trial that compares the efficacy of the H-Wave® Device with a commonly used TENS device and with a control group using a sham electrotherapy device [24]. A well-designed, high-quality trial will provide definable level of evidence for treatment efficacy, and provide a basis for evidence-based recommendation for or against utilization of the use of the H-Wave® device. The results for H-Wave® device, if positive, could significantly impact morbidity by providing a non-invasive, non-pharmacologic treatment for symptomatic relief, and reduce overall disability and health care costs associated with chronic low back pain.

## Methods/Design

### Participants

Subjects will be recruited from University and community practitioners, as well as through media advertisements. Enrollment and treatment will be conducted in clinical/laboratory facilities at the University of Utah’s Rocky Mountain Center for Occupational and Environmental Health.

Potential study subjects identified through the institutional review board (IRB) approved study marketing materials will be screened based on inclusion and exclusion criteria. If all criteria are met, they will be scheduled for a consent, randomization, and initial treatment.

Inclusion criteria for study eligibility includes: ages 18–65 with chronic low back pain (≥3 months duration; a current VAS pain rating ≥5/10; no radiating pain below the knee; ≥75% back or buttock pain rather than lower extremity pain; English speaking, and able to complete and tolerate treatment for the study period.

Exclusion criteria include: Prior home use of H-Wave® Device or TENS. Prior history of spinal fusion or failed spinal surgery syndrome. Laminectomy, laminotomy or discectomy within 12 months of enrollment. Diagnostic or interventional injections or any low back surgeries not mentioned above, including radiofrequency neuroablation within 6 months of enrollment. Current implanted cardiac demand pacemakers, defibrillators, cardiac pumps, spinal stimulators or other implanted electronic devices. Patients using personal home based electrical stimulation devices are excluded to reduce risk of blinding failure. Patients with other concomitant illnesses (e.g., malignancy, osteoporosis) which, in the opinion of the investigator, would preclude successful patient participation will also be excluded. Active psychiatric disorders will be excluded (e.g. use of antipsychotic medication, bipolar disorder, schizophrenia). Patients diagnosed with history of significant mood disorder will be excluded (e.g., depression or anxiety with adequate control would be acceptable). Patients currently are or who become pregnant will be excluded.

Consent will be obtained prior to randomization. Potential study subjects will be given a copy of the consent document and the document will be outlined in detail by a research team member. Potential study subjects will be given opportunities to ask questions prior to signing the consent form and enrolling in the study.

**Figure 1:**
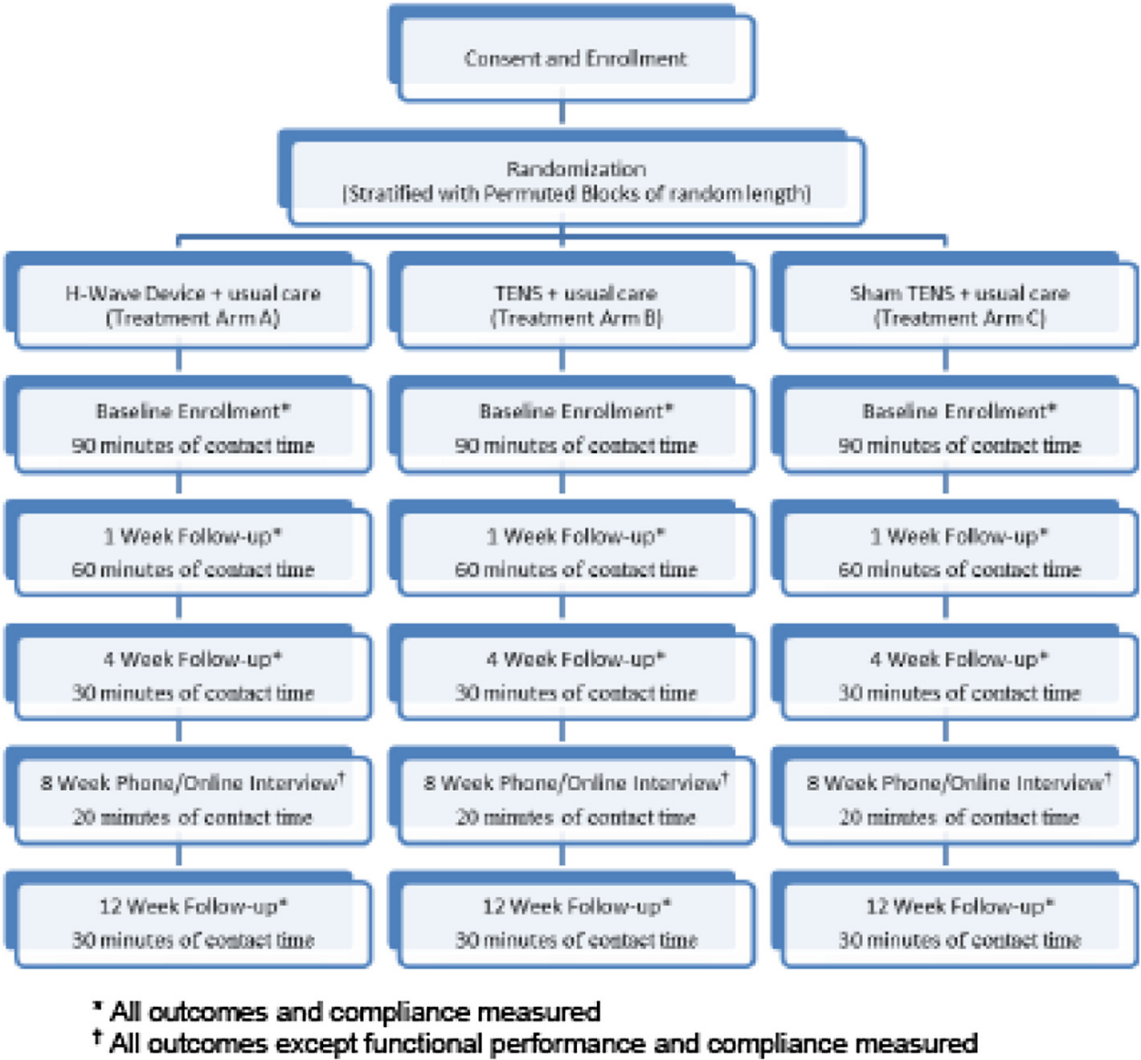
Trial design, procedures, stages and data collection.

This study has been approved through the University of Utah Institutional Review Board (IRB # 52918). Study participants will receive compensation for participation in this study (Figure 1).

### Randomization and allocation procedures

Subjects will be allocated through central, computerized randomization utilizing a stratified permuted-block randomization employing random block sizes. After confirming eligibility and completion of baseline data collection, the Study Coordinator will contact the designated investigator (MST) for allocation. He will consult the prepared block randomized list and the next allocation contained in sealed opaque envelope will be given to the treatment provider, while also recording the subject ID number to allow for ascertainment of compliance with treatment allocation. The three treatment arms are labeled using letters “L”, “W” and “E” and correspond to a labeled device. The names of particular devices will not be used. The treatment provider will open the envelope and provide the assigned treatment device to the participant. Block stratification will be performed for opioid use (current vs. past/never) and workers’ compensation claim (yes vs. no).

### Blinding

We will attempt to blind patients and assessors to achieve double blinding. Patients will not be told what electrotherapy techniques are being utilized in this study, only that there are two active electrotherapies out of the spectrum of electrotherapy devices and a sham group. All study materials will be void of the words H-Wave® or TENS. Patient blinding will be accomplished by providing all patients either a H-Wave® device, a fully operation TENS Unit modified to function within a H-Wave® device housing, or a sham electrotherapy device with minimally perceptible electrical current output modified to function within a H-Wave® device housing. Researchers providing the treatment are not blinded. Assessors are blinded and will not be present for the intervention. Participants will be instructed not to reveal or discuss the type of treatment characteristics they are experiencing.

Sham devices will appear to operate in the same exact way as a fully functional electrotherapy device, including battery drain and usage log. At maximal settings, weak electrical current will be flowing to the pads so that the patients may perceive the device as active treatment.

Additional blinding of subjects or treatment within this trial is not possible. The research team will be required to wear color-coded nametags indicating whether or not the researcher is blinded or non-blinded to avoid any confusion at the time of assessments. Efficacy of assessor blinding will be conducted at all onsite assessments by recording assessor’s opinion of treatment arm for each subject.

### Outcome measures

Outcome measures will be assessed at baseline, 1 week, 4 weeks, 8 weeks, and 12 weeks, with the primary outcome being at 4 weeks. Assessment will occur in the same manner and at the same time intervals for all treatment arms utilizing standardized methods. Treatment failure will be defined as if patient terminates assigned treatment arm for non-efficacy or election to pursue surgical or invasive procedure or other excluded cointerventions.

The primary outcome measure will be the Oswestry Disability Index (ODI). The ODI is widely used in low back pain intervention trials, and provides a measure of functional improvement The ODI was selected compared to a subjective pain rating [25-29]. Secondary outcome measures include Rowland Morris Instrument (0–24 scale of disability); VAS Pain score (0–100); physical activity levels objectively measured using accelerometers worn by participants during waking hours over during the entire 12 week study period; functional capacity evaluation including strength when pushing and pulling (3 repetitions lasting 5 seconds each of both pushing and pulling measuring both peak and average kg of force according to standard EvalTech Functional Systems protocol which utilizes a load cell to measure forces exerted); range of lumbar spine motion (up to participants maximum range of specific movement) in flexion, extension, lateral rotation; duration of time (minutes) sitting comfortably (subjective estimate made by participant during questionnaire); duration of time (minutes) standing comfortably (subjective estimate made by participant during questionnaire); duration (minutes) and distance (meters) walking comfortably (subjective estimate made by participant during questionnaire); summed VAS pain × days rating; days of impairment (days); lost workdays (days); time to exacerbation (days until increase 3/10 pain); rescue acetaminophen tablets consumed (% and number); NSAID discontinuation (%); opioid discontinuation (%). The pain VAS score is assessed immediately before and after treatment at weeks 0, 1, and daily scores pre and post home-treatment in a diary that the participant keeps (Table 1).

**Table 1:** Outcome measures

	
**Primary outcome measure:**	
	Oswestry Disability Index (ODI)
**Secondary measures:**	
a.	Rowland Morris Instrument (0–24 scale of disability)
b.	VAS Pain score (0–10)
c.	Physical activity levels (objectively measured using accelerometers)
d.	Functional evaluation including strength when pushing (peak and average kg of force)
e.	Functional evaluation including strength when pulling (peak and average kg of force)
f.	Comfortable (up to maximum baseline pain level) range of motion in flexion (degrees from neutral)
g.	Comfortable (up to maximum baseline pain level) range of motion in extension (degrees from neutral)
h.	Duration of time sitting comfortably (up to maximum baseline pain level) (minutes)
i.	Duration of time standing comfortably (up to maximum baseline pain level) (minutes)
j.	Duration and distance walking comfortably (up to maximum baseline pain level) (minutes and meters)
k.	Summed VAS pain × days rating
l.	Days of impairment (days)
m.	Lost workdays (days)
n.	Time to exacerbation (days until increase 3/10 pain)
o.	Rescue acetaminophen tablets consumed (% and number)
p.	NSAID discontinuation (%)
q.	Opioid discontinuation (%)

**Figure 2:**
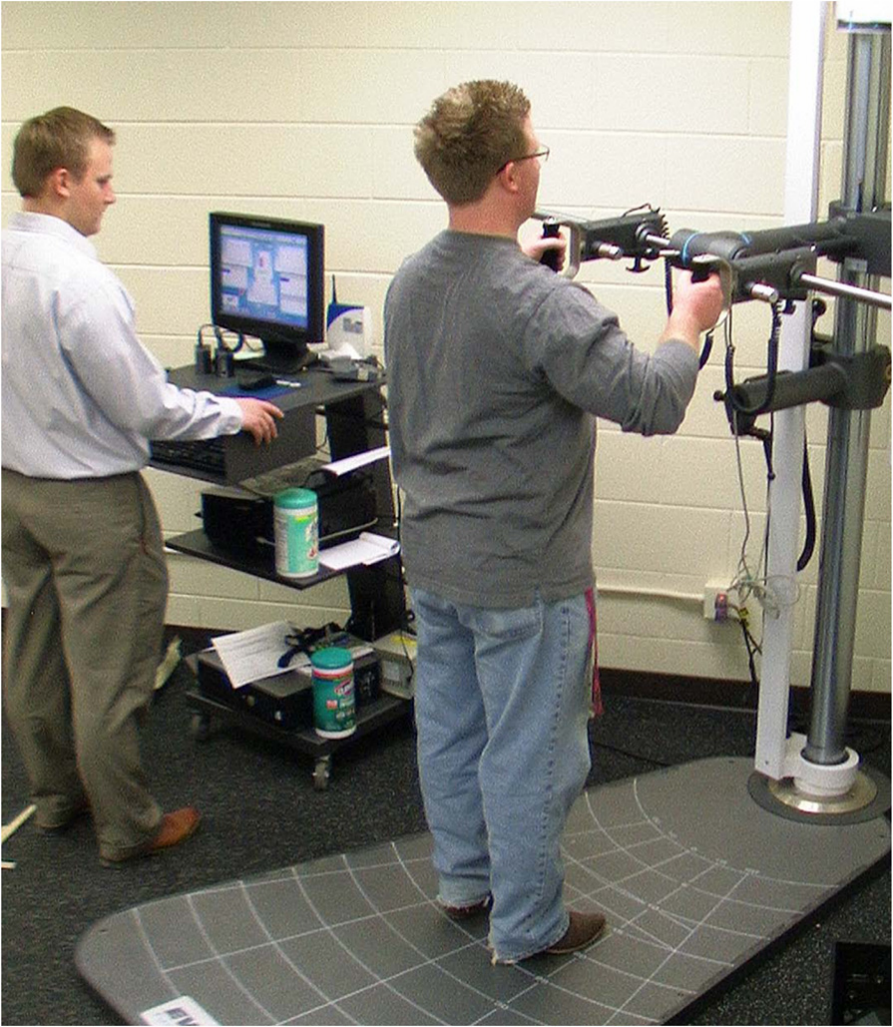
Shoulder height push assessment using BTE EvalTech system.

Functional assessments will be completed using a standardized protocol on a BTE EvalTech system. Standard protocols for Lumbar Flexion/Extension, Lumbar Lateral Flexion, Shoulder Height Push and Pull, Cart Height Push and Pull are used. Researchers performing assessments are trained to perform all assessments exactly the same following the BTE EvalTtech protocol (Figure 2).

Subjective outcomes data will be collected using computerized data collection tools utilizing Filemaker Pro™ database management to assure complete and accurate data collection. Data will be collected at enrollment and subsequent treatments. Data after treatments end will be collected in person using the same tools for every participant study by a blinded assessor.

### Assessments

Timing of assessments is identical in each arm. Assessments will be conducted by blinded assessors. Enrollment, randomization and initial treatment will occur at the time of enrollment. All participants will return 1 week after initial treatment for assessment of subject ability to correctly use their assigned device; have questions answered about the protocol, and to complete week 1 assessment. Subjective assessments will be measured at baseline, 1 week, 4 weeks, 8 weeks, and 12 weeks. Objective assessments will be measured pre and post-treatment at baseline and post-treatment at week 1, week 4, and week 12. No treatments are provided by the researchers after week 1, although patients will be performing home treatments per protocol for the entire 12 week period. Compliance will be assessed using digital time data logs recorded automatically by each device and diaries kept by participants. Participants in each arm will have identical and comparable contact time with providers and assessors to minimize treatment bias. All participants will be contacted weekly by a blinded assessor via telephone to encourage compliance and use of daily treatment diaries.

At each assessment, all participants receive the same assessment, including questionnaire, physical functioning assessment form, and recording of accelerometer use. All measures will be assessed using computerized data collection methods at baseline and each follow-up, except at the 8-week follow-up where outcomes will be assessed over the phone. There are four objective measures which are assessed via electronic device (Actigraph GT3X + Accelerometers and EvalTech functional testing equipment manufactured by BTE Technologies). The accelerometers provide physical activity levels on a per minute basis and store up to 40 days of data. These accelerometers have demonstrated validity and reliability in a variety of settings [30-38]. Functional strength and lumbar range of motion will be captured using EvalTech functional evaluation tools. These include fully modifiable load cells allowing different heights, widths, and orientations, as well as electronic range of motion devices for adequate capture. All trials will be performed 3 times. Pushing and pulling strength will be three trials each with exertions for 5 seconds and a 20 second break between trials. These will be at both shoulder height and cart height (approximately 49 inches). There will be a minimum of 6 trials (3 push and 3 pull at both shoulder height and cart height). Peak and average forces will be recorded for each trial.

### Treatment protocols

Subjects will be allocated to one of the three arms. Each treatment arm will have identical numbers of visits and contact time. Each arm will receive the uniform instructions for using of their assigned electrotherapy device. Patients will be instructed to use their assigned device daily upon waking and before bedtime, and up to two additional treatments during the day as needed (e.g., 4 1-hour treatment sessions). Providers will place electrodes and activate the device as per the protocol for each arm. Patients will be told that it is possible to not immediately feel effects of electrical treatment due to current levels that do not meet sensory thresholds. Patients will return after 1 week for reevaluation on electrode placement and ability to achieve the required self-administered stimulation levels. Providers are trained in standardized fashion to educate each participant on their assigned therapy.

The H-Wave® device delivers electrical stimulation through the placement of 4 electrode pads in a rectangular geometric pattern at the circumference of the low back pain nidus. Patients will be instructed on optimal electrode placement for home use. The H-Wave® Device is controlled by the patient after instruction from a therapist or practitioner. The stimulation parameters in terms of pulse duration and frequency are proprietary for the manufacturer and thus are not included in this protocol. However, the maximal intensity offered by the device is the goal of the treatment intensity. Patients are instructed to achieve the highest intensity possible in graduated fashion. Lower intensities are accepted if the patient cannot comfortably reach the maximal device intensity and will be noted on diary logs. There are controls for intensity and frequency, which the patient manipulates according to specific instructions and tolerances. Patients in this arm will be required to reach near maximal intensity on low frequency. The maximal intensity may, in rare cases, induce transient muscular contractions. Patients are instructed not to increase the intensity to a painful level. The treatment is then self-administered for the duration of the study.

There are no accepted standardized protocols found in the literature for TENs treatment of chronic low back pain. There are no trials that demonstrate maximum efficacy of pulse duration, wave type, or frequency. Therefore, the TENS units in this study allow adjustment of amplitude (intensity) only at a fixed frequency of 100 Hz with steady state pulsing. Modulation and pulse modes will be disabled in order to maintain uniformity of electrotherapy protocols. Pad placement will be directed by the provider to provide maximal clinical efficacy. The device is two channel TENS and will be administered by the patient after training by the provider.

The sham or control device will appear to operate in the same exact way as a fully functional TENS device, including battery drain and usage log. Subtherapeutic electrical current will be flowing to the pads so that the patient may perceive it as an active therapy. They will be instructed to use the device for the same duration as active treatments, daily upon waking and before bedtime, and up to two additional treatments during the day as needed (e.g., 4 1-hour treatment sessions). There is no quality evidence that subtherapeutic electrical current provides any clinical benefit over no electrotherapy effectiveness for LBP, however this will likely blind the patients because none of them have ever experienced the H-Wave Device. Blinding of patients is achieved as none will have previously experienced H-Wave, home based TENS therapy, or subtherapeutic electrotherapy. Further, patients are kept blind as to which electrotherapy devices out of the myriad devices on the market, are included in the study and therefore have few preconceived notions to their assigned device.

### Compliance

Compliance will be assessed by device logs and diaries. Each device will be capable of recording treatment applications and duration, and will be downloaded electronically at each in-person assessment. Total treatment time of 3.5 hours per week will be set as the threshold for compliance. If VAS Pain drops to a daily average of less than or equal to 2 out of 10, patients will be considered in compliance regardless of the number of treatments completed on that day. Patients will be called once per week to promote compliance and answer any questions.

### Discontinuation

Subjects will be encouraged to continue to follow-up throughout the study. There are no penalties for early dropouts other than the amount of incentive earned is reduced if the entire follow-up period is not completed. Each device (H-Wave®, TENS) has very low adverse effect profile. Although unexpected, those developing an adverse event will be reported to the IRB.

### Usual care and co-interventions

Study participants will be asked to avoid significant changes in medical/healthcare management of their LBP during the study period. All participants will be allowed to pursue usual care with their own personal treating physician. No other treatment other than the assigned electrotherapy intervention will be provided by the trial researchers. Usual care will be limited to non-invasive therapies, including medications such as NSAIDS (prescription or over the counter) and opioids, physical therapy, manipulation, stretching and aerobic exercise. Self-applications of heat or ice are also acceptable. All usual care medications and interventions will be recorded.

### Interventions not allowed

Any lumbosacral invasive procedure such as discectomy, microdiscetomy, laminectomy, lumbar fusion, disc desiccation by any chemical or thermal means. No injections into the lumbosacral area other than localized trigger point without the use of glucocorticoids. No other electrical therapies (PENS, Interferential therapy). No use of oral or systemic glucocorticosteroids. TENS and H-Wave® are not allowed except in the arms assigned to those treatments. Patients are asked about any additional treatments received at each assessment to determine continued eligibility.

### Statistical approach

#### Statistical power/Sample size

Sample size determination is based on the comparisons of the study arms for the primary outcome variable, ODI. In previous studies of low back pain patients , the baseline ODI values for one study group were mean (SD) of 60.2 (15.0) and for a second study group were 59.9 (14.4) [39,40]. The total score for the ODI is a percentage, ranging from 0 to 100. It is suggested that the reliability of the ODI requires a minimum detectable change of at least 10 points, as any smaller difference may be attributable to error in the measurement [25]. Assuming equality of the study arms at baseline, which can be assured by adjusting for baseline ODI in the regression model, and a minimal detectable effect size of a 10 point difference on the post-treatment value, sample size is based on a comparison of mean (SD) of 60 (15) vs. 50 (15), a 17% relative difference post treatment, using a two-sided comparison with alpha of 0.025 to allow for multiplicity adjustment. Under these assumptions, a sample size of n = 43 subjects per study arm is required to achieve 80% power. However, it is expected that the stimulation arms will produce at least a 25% relative improvement over the control arm. Assuming 60 (15) vs. 45 (15), a 25% relative difference, using a two-sided alpha 0.025 comparison, a sample size of n = 20 subjects per study arm is required for 80% power, or n = 25 subjects for 90% power. Allowing for a 20% dropout rate, a sample size of n = 38 subjects will be enrolled in each study arm to provide n = 25 evaluable subjects for data analysis.

### Statistical analysis

#### Primary analysis

The primary analysis is the comparison of the three study arms on the primary outcome, Oswestry Disability Index (ODI). Given that repeated measurements are collected, a mixed effects linear regression model will be fitted with an unstructured covariance matrix to account for lack of independence introduced by the repeated measurements. In this model, the repeated measurements are nested within study subject (weeks 0, or baseline, and weeks 1, 4, 8, and 12). The primary predictor is study group. A time variable will be included as a covariate, since visits will not always be at the planned intervals. Other covariates will be assessed for possible confounding in the relationship between treatment arm and outcome. It is likely that the pattern of dropouts will vary between the study groups, where the study interventions that are most effective having the dropouts later on in the follow-up, while the intervention least effective to have dropouts early on. The analysis will emphasize the 4-week time point as the primary end point, which ends the intensive intervention period. At 4 weeks compliance to the protocol and complete follow-up should be at a maximum. The remaining time to 12 weeks will measure long term effectiveness and acceptance of the study interventions.

#### Secondary analyses

The changes from baseline to each follow-up visit in the pain VAS (the main secondary endpoint) will be analyzed using the same longitudinal model described above for the Oswestry score. Key secondary comparisons will include the three pair wise comparisons between the treatment groups in the mean change in the Roland Morris Instrument, VAS, strength, and flexibility measures at the different time points.,

The changes to 12 weeks will evaluate the persistence of treatment effects of the interventions to that time; changes to 4 weeks evaluate treatment effects at the completion of the intensive intervention period, and the slopes from weeks 4 to 12 evaluate changes in the treatment effects after 4 weeks. The null hypothesis is that there is no difference between the three treatment arms at 12 weeks.

#### Intent-to-treat analysis

These data will be analyzed using an intent-to-treat analysis. As H-Wave® Device is not widely utilized in study catchment area it is doubtful that individuals will inadvertently crossover to that arm from the controls. However, that possibility will be both discouraged and tracked.

#### Multiplicity

A multiplicity adjustment will be made for the comparison of the primary outcome variable, Oswestry Disability Index (ODI). The two-sided p values from the hypothesis tests will be adjusted for two comparisons using the Hochberg procedure [41]. With that procedure, the smaller of the two p values is compared against alpha = 0.025 and the larger p value is compared against alpha = 0.05. This maintains the overall alpha at 0.05 for the effectiveness win strategy evaluation.

#### Co-variates

Age, gender, tobacco, undiagnosed anxiety, undiagnosed depression, past back pain history, prior and current NSAID use, rescue acetaminophen use.

#### Post-hoc analyses

Post-hoc analyses will be conducted to attempt to ascertain what factors predicted better responders among the two treatment arms. Potential prognostic factors include gender, tobacco, anxiety, depression, (age and past back pain history will be assessed for residual confounding, but controlled in study design), belief in effectiveness of the treatments, NSAID use, rescue acetaminophen use.

#### Other considerations

The primary and main secondary analyses will be performed using an intent-to-treat strategy in which patients are analyzed based on their randomized assignment irrespective of their compliance to the treatment protocol. Formal interim analyses of efficacy will not be conducted. However, the operational aspects of the study (recruitment, retention, adherence rates, and rates of missed visits) will be monitored as the study progresses so that corrective action may be taken to address any unexpected difficulties. Monitoring for safety will be conducted throughout the study, investigating all possible unanticipated safety events, including increased pain and discomfort which are the two most likely types of events to occur. These treatments are widely used and are considered safe with low rates of adverse effects. Efforts will be made to minimize the rates of missing data, including telephone follow-up of missed visits. The restricted maximum likelihood procedure of the primary analysis produces approximately unbiased estimates of the treatment effects so long as data are missing at random [42] and is less sensitive to missing data than analyses restricting to complete data [43]. In sensitivity analyses, multiple imputation incorporating baseline and follow-up predictors of outcome [44] will be used to assess the effect of potential violations of the missing at random assumption.

## Discussion

Chronic low back pain is a significant issue, resulting in significant costs, lost productivity, and morbidity [1-6]. Therefore, even small improvements in treatment efficacy, particularly for treatments with few side effects, can have a meaningful impact on improving LBP. While there have been some studies assessing the therapeutic effectiveness of TENS, the effect generally is relatively small improvements in chronic LBP as compared to sham treatment. The use of two comparison arms in this study, both TENS and sham treatment, allows for comparisons of experimental device with both TENS as well as sham treatment.

Blinding within this study is a significant strength. All devices are identical in appearance, weight, display, charge time, and sound. It is anticipated that complete blinding of the assessors and patients will be achieved. Care will be taken to limit contact between participants, e.g. schedule participants one at a time so they are not waiting in the same room together. All treatment questions will be directed to the interventionalists, as to maintain blinding of the assessors. Additionally, this protocol outlines concealing the treatment allocation by using opaque envelopes that appear identical. The utilization of a complex randomization schedule is another strength that may ensure equality of treatment groups at baseline. The intent-to-treat analysis may account for unequal dropout between groups and any contamination that may occur.

Potential weaknesses include the possibility of high dropout in one or more treatment arm. To combat this, we are offering incentives and encouraging participants to continue for the entire duration of the study. There is the possibility of a randomization failure, which cannot be overcome. Additionally, due to the nature of the treatment, specifically the differences between the experimental treatment and the TENS treatment, it was not feasible to blind the interventionalist. To address this all interventionalists will undergo strict training and re-standardization based on the treatment protocol. This training will stress treating all patients similarly with regards to demeanor and contact time, regardless of their randomized treatment arm.

## Endnotes

^a^ Based on the ACOEM criteria, this study protocol is intended to provide a high-quality score (Randomization – 1, Blinded Allocation – 1, Baseline Comparability – 1, Patient blinding – 1, Interventionist blinding – 0, Assessor Blinding – 1, Control of co-interventions – 0.5, Compliance – 1.0, Drop Out < 20% – 1, Timing of assessments – 1, ITT analysis – 1 (Total score 9.5/11) High-quality ≥ 8.0.

## Competing interests

The authors declare they do not have any competing interests.

## Authors’ contributions

All listed authors contributed to the conception and design of the study. All authors were involved in drafting the manuscript and read and approved the final manuscript.

## Pre-publication history

The pre-publication history for this paper can be accessed here:

http://www.biomedcentral.com/1471-2474/14/117/prepub
